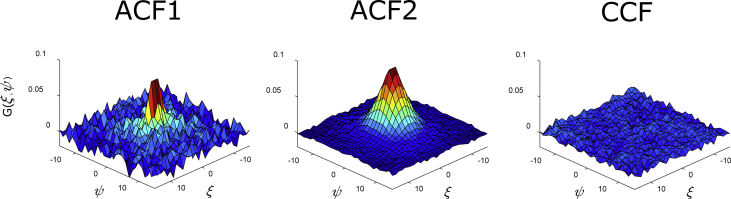# Fluorescence microscopy data for quantitative mobility and interaction analysis of proteins in living cells

**DOI:** 10.1016/j.dib.2020.105348

**Published:** 2020-02-28

**Authors:** Veerle Lemmens, Keerthana Ramanathan, Jelle Hendrix

**Affiliations:** Hasselt University, Advanced Optical Microscopy Centre and Biomedical Research Institute, Dynamic Bioimaging Lab, Diepenbeek, Belgium

**Keywords:** Confocal laser scanning microscopy, Fluorescence fluctuation spectroscopy, Raster image correlation spectroscopy, Diffusion, Multicolor interaction analysis

## Abstract

The data provided with this paper are image series of slowly diffusing GlyRa3 molecules, linked to either eGFP or mCherry fluorescent proteins, at the membrane of HEK cells, acquired on a Zeiss LSM880 confocal laser scanning microscope. Raster spectral image cross-correlation spectroscopy (RSICS) is applied to the data, a technique that exploits intensity fluctuations in confocal image series recorded using a spectral detector to study the diffusion and concentration of molecules, and interactions between them. First, spectral filters are created from reference image series containing GlyRa3 labeled with a single fluorophore. Once experimental data containing GlyRa3 labeled with both fluorophores is acquired, single images are either autocorrelated, or the cross-correlation is calculated between two images, each one containing the data for eGFP or mCherry labeled GyRa 3. Data is then fit with a one-component model assuming a two-dimensional Gaussian point spread function to obtain the diffusion coefficient, *D*, and average number of molecules in the focus, *N*. The software package PAM is used to analyze all the acquired data. The data can be used as a reference for artifact-free two-color ccRICS that contains slowly diffusing interacting molecules. Additionally, the analysis workflow described in this paper helps researchers avoid common errors during a RICS experiment.

**Table:** Specifications Table

Subject	Biophysics
Specific subject area	Optical imaging
Type of data	Image Image series
How data were acquired	ZEISS LSM880 with 34-Channel Quasar detector
Data format	Raw. czi ZEISS microscopy time series Filtered.tif files Analyzed output data
Parameters for data collection	Data were collected using imaging parameters optimal for RICS analysis [1]
Description of data collection	Data is acquired at the membrane of HEK293 cells which were transiently co-transfected to express the green and red proteins of interest. For each experiment, green-only and red-only pure-species reference data was acquired in the same experimental conditions.
Data source location	Biomedical research institute, Hasselt University, Diepenbeek, Belgium, 50°55′44.4″N 5°23′32.7″E
Data accessibility	With the article
Related research article	Waldemar Schrimpf, Veerle Lemmens, Nick Smisdom, Marcel Ameloot, Don C. Lamb, Jelle Hendrix. Crosstalk-free multicolor RICS using spectral weighting, Methods, https://doi.org/10.1016/j.ymeth.2018.01.022

**Table:** 

**Value of the Data** • The data can be used as a reference for artifact-free two-color ccRICS analysis of a sample containing slowly diffusing (D = 0.1 μm^2^/s) interacting molecules. • These data are useful for researchers who are interested in studying molecular mobility, interactions and binding ratios (stoichiometry), and who are setting up and optimizing RICS experiments. • The data and associated analysis workflow (data acquisition optimization, spectral filtering, image masking, model fitting) help researchers to avoid possible pitfalls in quantitative RICS experiments.

## 1 Data description

The raw data (GlyR.czi, REFeGFP.czi and REFmcherry.czi) are confocal image series acquired at 37 °C at the cell membrane of live HEK293 cells expressing one or two types of fluorescent protein labeled glycine receptors (GlyRa3-eGFP and/or GlyRa3-mCherry). The .tif files (GlyR_REFeGFP.tif and GlyR_REFmCherry.tif) are the spectrally weighted versions of the raw data. The .miacor files are the calculated autocorrelation (GlyR_ACF1. miacor and GlyR_ACF2. miacor) and cross-correlation (GlyR_CCF.miacor) functions from the spectrally filtered data, that can be opened in the analysis software. A stepwise RSICS protocol is explained in Fig. 1. Spectral patterns of the reference data and its generated spectral filter is described in Fig. 2. The auto- and cross-correlation functions of the membrane protein are shown in Fig. 3 with additional 3D correlations in Fig S1. Graphs to interpret their interaction are shown in Fig. 4 and for comparison a case of non-interacting membrane proteins is added in Fig S2 with 3D correlations in Fig S3.

**Fig. 1:**
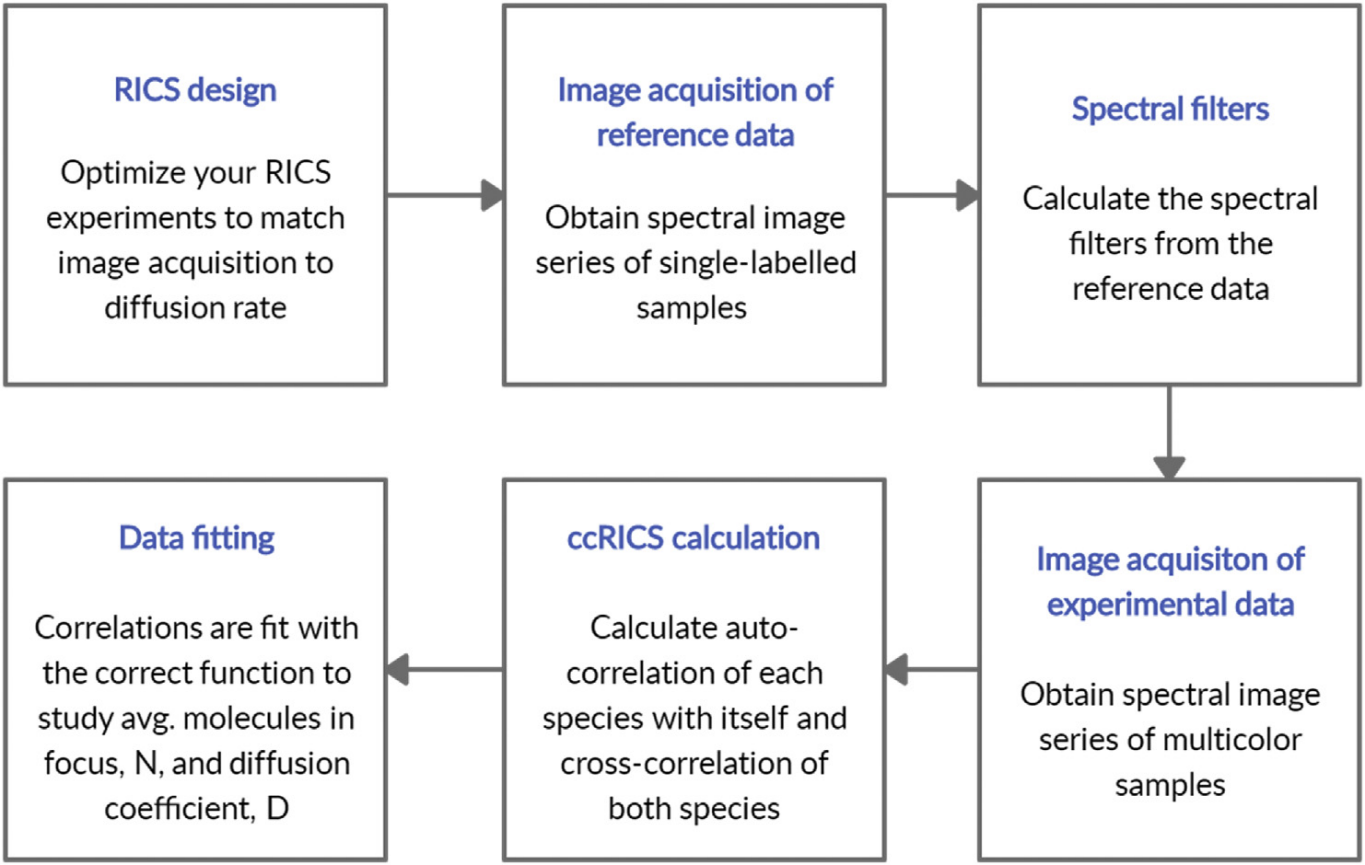
Flowchart explaining RSICS analysis.

**Fig. 2:**
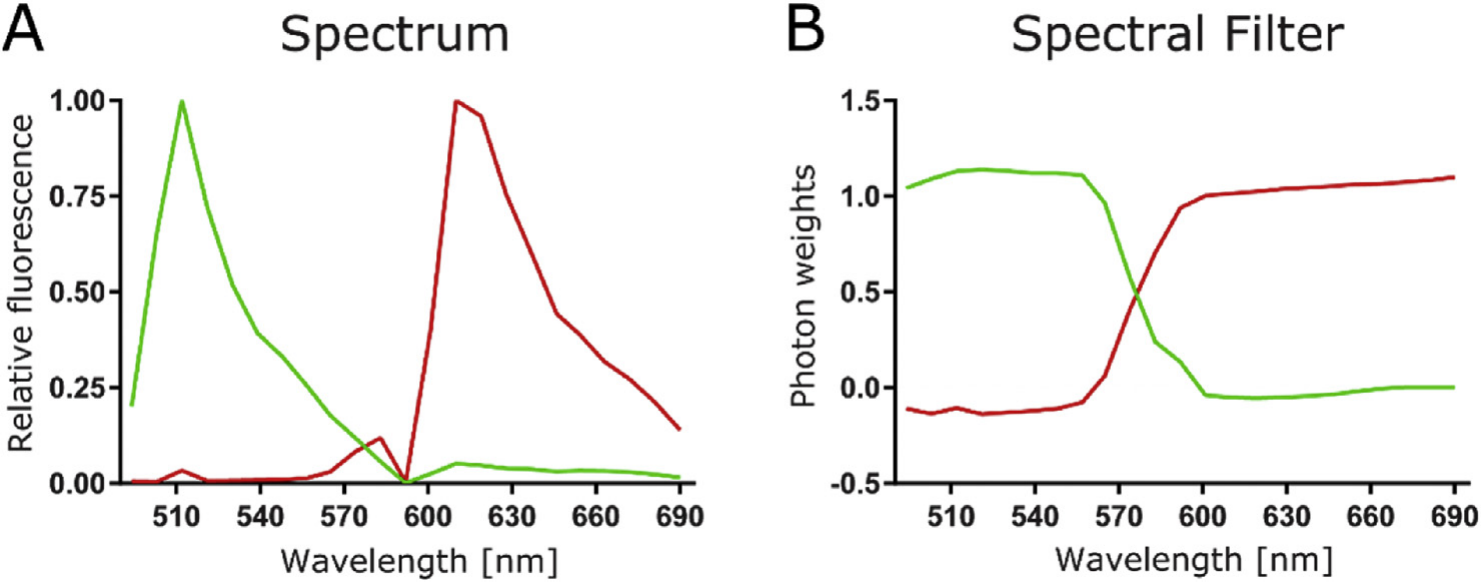
Emission spectrum pattern and generated spectral filter. A) Emission spectra of the single-color reference data eGFP (left, green) and mCherry (right, red) from the relative frequency of photons detected in the different spectral bins. B) Spectral filters generated with the spectral pattern in A. (For interpretation of the references to color in this figure legend, the reader is referred to the Web version of this article.)

**Fig. 3:**
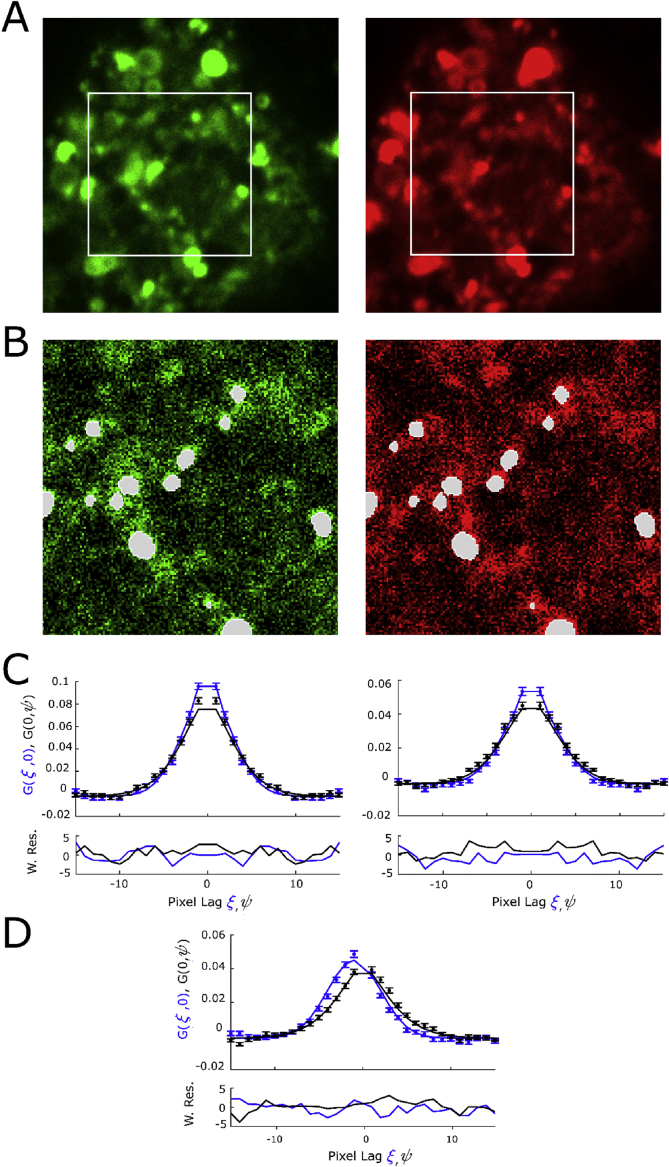
Auto- and cross-correlation analysis of diffusing membrane protein. A) Average images from a 60-frame image series of a HEK293 cell co-expressing eGFP- and mCherry-tagged GlyRa3 weighted towards eGFP (left) and mCherry species (right). The white rectangle represents the selected region-of-interest to exclude the region outside the cell. B) First image frame corrected for slow cell movement showing pixel regions in white for exclusion, determined by intensity-thresholding in both the left and right image on a per image frame base using a 3 × 3 median filter for masking. C + D) Autocorrelation and cross-correlation functions were calculated from the preprocessed ROIs using the arbitrary region algorithm. In all graphs, only the (*ξ*, 0) and (0,*Ψ*) sections are shown to allow plotting the data and fit function on one graph. The bottom graphs display the weighted residuals for the fit in the top graphs. Additional 3D correlations are shown in Fig S1.

**Fig. 4:**
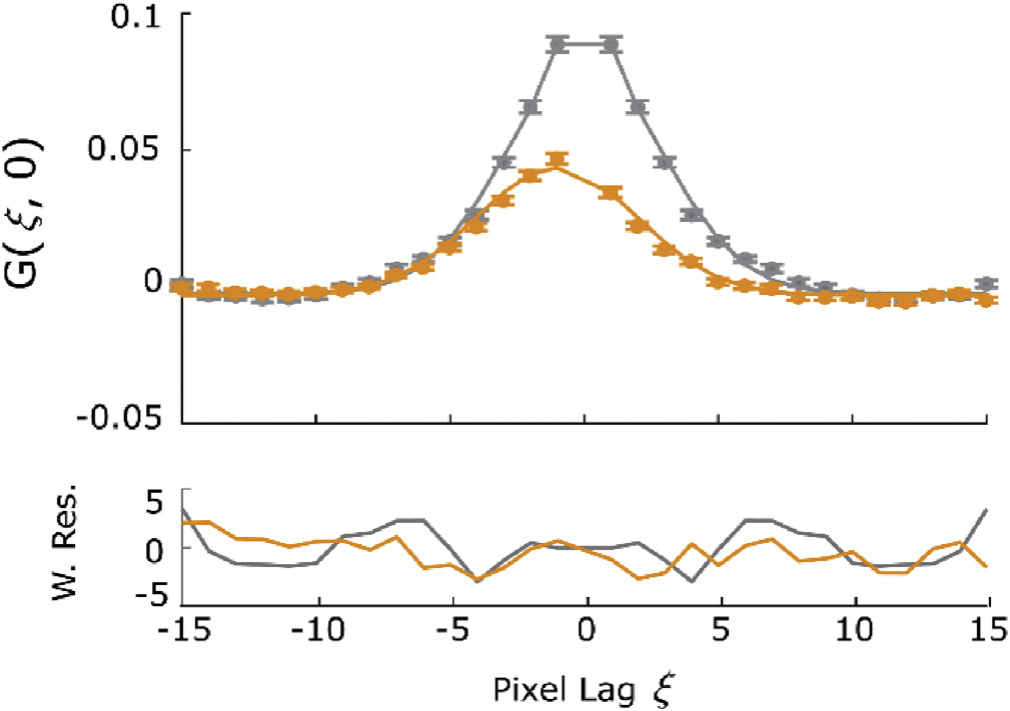
Comparison of the auto- (grey) and cross-correlation function (orange) to analyze interaction. The bottom graphs display the weighted residuals for the fit in the top graphs. Only the (*ξ*, 0) correlation function sections are shown to allow plotting auto and cross-correlations on one graph. (For interpretation of the references to color in this figure legend, the reader is referred to the Web version of this article.)

## 2 Experimental design, materials, and methods

### 2.1 Image acquisition

A ZEISS LSM880 laser scanning microscope and Plan-Apochromat 63x/1.4 Oil DIC M27 oil objective was used to image the HEK293 cells. For each measurement at least 60 frames were acquired at the cell membrane. Images contain 256 × 256 pixels^2^ with 50-nm pixel size to have multiple pixels in the point-spread function. Pixel dwell, line and image times were 8.19 μs, 4.92 ms and 1.26 s, respectively. Depending on the diffusion constant range that needs to be quantified, the optimal imaging settings can be predicted [1], although care has to be taken that the frame times is not too long, to avoid significant frame-to-frame cell or organelle movement that will negatively influence any further image processing. The eGFP species were excited with a 488-nm argon-ion laser (1 μW in the sample) and mCherry species with a 594-nm HeNe laser (6 μW in the sample). Detection of fluorescence emission light occurred in photon-counting mode in 23 spectral bins ranging from 490 nm to 695 nm with the ZEISS 34-Channel Quasar detector. The time series were saved as .czi files within the ZEISS software.

All analyses of the experimental data were performed in the software package PAM [2]. The software is available as source code, requiring MATLAB to run, or as pre-compiled standalone distributions for Windows or MacOS at http://www.cup.uni-muenchen.de/pc/lamb/software/pam.html or hosted in Git repositories under http://www.gitlab.com/PAM-PIE/PAM and http://www.gitlab.com/PAM-PIE/PAMcompiled.

A flowchart for the applied ccRICS, analysis using spectral weighting is shown in Fig. 1. The acquired data is spectrally filtered before calculation of RICS auto- and cross-correlation functions. Finally, data is fitted with a 2D fit function to obtain protein concentrations, diffusion coefficient and interaction.

### 2.2 Spectral filtering

The spectra of the experimental single-color references data (REFeGFP.czi and REFmCherry.czi) are shown in Fig. 2A. While the maximum emission peaks for eGFP (508-517-nm bin) and mCherry (606-614-nm bin) are well-separated, photons from eGFP can be observed in most mCherry bins due to spectral crosstalk. The dip around 590 nm is due to residual 594-nm laser light being blocked in this spectral bin. Using the spectral pattern of the pure reference species, a spectral filter function was generated as explained in Ref. [3]. Next, different spectral images are combined prior to correlation analysis. Specifically, we multiply each spectral image with its corresponding eGFP or mCherry spectral filter value, and then sum all weighted images (Fig. 2B). When applied to the image series of interest (GlyR.czi) this results in two .tif files, one weighted towards the green (GlyR_REFeGFP.tif) and one towards the red species (GlyR_REFmCherry.tif).

### 2.3 ccRICS analysis

Prior to the auto- and cross-correlation analysis as originally described in Ref. [[4], [5], [6]] the newly generated weighted .tif files (GlyR_REFeGFP.tif and GlyR_REFmCherry.tif) were further processed. First, a square region-of-interest was selected to exclude pixels outside the cell (Fig. 3A). Next, slow processes such as cell movement were removed from the image series using a moving average correction with a phase-conserving (from frame-1 to frame+1) 3-frame window according to Ref. [6,7]. The fluctuation amplitude bias introduced by the moving average correction is corrected according to Ref. [8]. In addition, membrane protein aggregates were excluded using intensity thresholding. Practically, and as detailed in the original paper on RICS in arbitrary regions-of-interest (ARICS) [8], the center pixel in a small (∼5 × 5 pixels^2^) region-of-interest was excluded from further analysis if the mean intensity of the small ROI was >3 times the mean intensity of the complete photobleaching-corrected image series. Furthermore, the excluded-pixels mask was spatially filtered by a 3 × 3 median filter (Fig. 3B).

The autocorrelation $\left(I_{1}=\;I_{2}\right)$ and cross-correlation function $\left(I_{1}\neq \;I_{2}\right)$ (Fig. 3C and D) were calculated using the ARICS algorithm [8],

$$G\left(\xi ,\psi \right)=\;\frac{\langle \delta I_{1}\left(x,\;y\right)\cdot \delta I_{2}\left(x+\xi \;,\;y+\psi \right)\rangle }{\langle I_{1}\rangle \cdot \langle I_{2}\rangle },$$

in which ξ and ψ are the spatial lags in pixels, x and y denote the pixel coordinates. The angled brackets represent the average over all included pixels in the image. δI_i_ is the fluctuation in signal intensity and is calculated according to $\text{δ}I_{i}\left(x,y\right)=\;I_{i}\left(x,y\right)-\langle I_{i}\rangle$. The obtained functions were saved as .miacor files and can be viewed using the PAM software.

### 2.4 Data fitting

The auto- and cross-correlation functions (GlyR_ACF1. miacor, GlyR_ACF2. miacor, GlyR_CCF.miacor) were fitted with a one-component model assuming a two-dimensional Gaussian point spread function to obtain the diffusion coefficient, *D,* and average number of molecules in the focus, N.

$$G\left(\xi ,\;\psi \right)=\frac{\gamma }{N}{\left(1+\frac{4D\left|\xi \tau_{p}+\psi \tau_{l}\right|}{\omega_{r}^{2}}\right)}^{-1}\;\text{exp}\left(-\frac{\delta r^{2}\left({\left(\xi -s_{x}\right)}^{2}+{\left(\psi -s_{y}\right)}^{2}\right)}{\omega_{r}^{2}+4D\left|\xi \tau_{p}+\psi \tau_{l}\right|}\right)$$

here γ is the shape factor for a 2D Gaussian and equals 2^−3/2^. The shift of the PSF between the green and red laser needed for fitting the cross-correlation function is represented by $s_{x}$ for the x-direction and by $s_{y}$ for the y-direction.

To quantify the interaction between the green and red species, typically the G(0) value of the cross-correlation function is compared to the G(0) value of the autocorrelation, with a high value of this ratio meaning a strong interaction. In Fig. 4, the fast axis of the autocorrelation function of the species weighted towards eGFP is compared to the fast axis of the cross-correlation function. A case where there is limited or no interaction between membrane proteins is shown in the supporting Fig. S2 and Fig. S3.

## Figures and Tables

**figs1:**
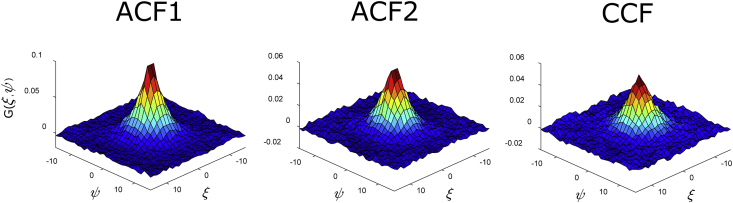


**figs2:**
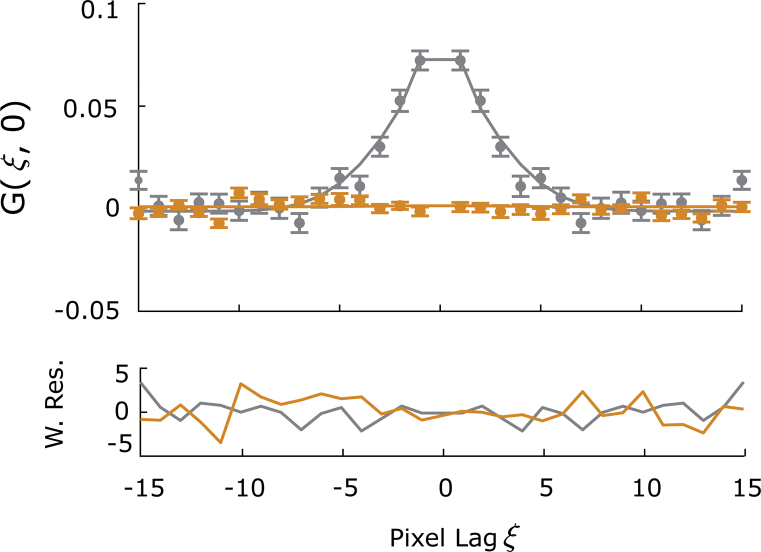


**figs3:**